# Some Practical Hints on the Fitting of Spectacles

**Published:** 1881-08

**Authors:** Frank W. Abbott


### BUFFALO

### Medical and Surgical Journal.

Vol. XXI. AUGUST, 1881. No. 1.

### Original Communications.

### Some Practical Hints on the Fitting of Spectacles.

By Frank W. Abbott, M. D.

There is perhaps nothing which everybody uses who reaches the declivity of life, and many of the most intelligent in early years, that is used with so little appreciation of the causes for their need and the principles which govern their correct adjustment, as spectacles. Fortunately the healthy eye is a patient and long-suffering member and adapts itself to adverse circumstances with great facility.

A normal condition of vision obtains when, without conscious effort, a clearly outlined picture of an object at any distance falls upon the yellow spot of both retinas. Spectacles fulfill their purpose when with their assistance a perfect picture of the observed object falls upon the retina without conscious effect, and the eye needs glasses when, with healthy tunics and humors, a sharply outlined picture of the observed object does not fall upon the retina at all or only with conscious effort. The ordinary conditions which prevent this desired state of things are called errors of refraction, as you all know, and are presbyopia, myopia, hypermetropia, and astigmatism. As presbyopia affects everybody who reaches the age of fifty years, and as almost every civilized person of that age wears glasses more or less, we will consider this error of refraction first.

Our ordinary book and newspaper print is legible at a distance of about twelve or fourteen inches from the eye, and this the man less than forty years of age can read with ease and comfort. Soon after this age, however, he begins usually to grumble a little at the poor type that printers use now-a-days, and is particular about the light which he uses. It must be pretty strong and fall squarely upon the page or else it doesn't suit him. Perhaps as characteristic a motion as any that he makes now is when he looks at his watch to tell the time of day. When he first pulls out his watch he involuntarily brings it up within eight or ten inches of his face, as he used to years ago, but the figures are indistinct, and with a half surprised, half-impatient gesture throws his head back and pushes his watch a few inches farther away, and soon sees it plainly. It is very likely that at this time any hint by friends that his sight is not what it once was will be indignantly resented, for he says what is very true that he can see off as far as ever he could in his life, and may challenge his friend to a competition in reading a distant sign or placard, and show that in this respect his natural powers are undiminished.

Now, what change has taken place in his eyes to make the reading of his evening paper irksome, instead of easy, as it was formerly?

It is strange that such a theory as that the cornea becomes flattened ever prevailed, for you see at once that a change of this kind would have affected distant vision as well as near. I may be going over ground well known to you all when I give the correct theory of the refractive processes of the eye, but I must make my paper logical at the expense of telling a twice-told tale.

Dr. Moore says that Ophthalmologists love long words. Grant it. But our long words mean something and they have the same sharp definite meaning every time, and when you once know the lingo, as a revered Buffalo D. D. forcibly puts it, these long words give a precision and accuracy to your statements, which a long circumlocution in short words fails to afford.

Now, the first long word which I want to use, is emmetropia and its adjective emmetropic. An emmetropic eye is a normally shaped eye, in which at perfect rest distant objects are distinctly seen. Emmetropia from en. in metron measure, ops eye, means this condition of eye, in which the different parts are so in measure that vision for distance takes place without effort, for a perfect picture of the observed object as made by the lens, is printed directly on the lens, for the retina is directly at the principal focus of the lens. Now, when this emmetropic eye looks at anything near by, of course if no change occurs in it, a perfect picture is not made on the retina, for with the change in the position of the object comes a change in the position of the focus of the lens in relation to it.

Here the function of the ciliary muscle steps in. As the object approaches, through the influence of the ciliary muscle, the lens becomes stronger and the focus still remains directly at the retina, a perfect picture is formed upon it and distinct vision results. The eye accommodates itself to any distance of the object we wish to see, and this faculty is hence called accommodation in our lingo. Now, our theory of the refractive processes of the eye is established. The emmetropic or normally-shaped and constructed eye sees perfectly at a distance when at rest, and accommodates itself for near vision by making the lens more convex or stronger.

It is probably superfluous to say that this accommodation becomes an automatic action before we are old enough to be self-conscious, and up to middle life we look from distant things too near and the eye adjusts itself to them, so as to make vision perfect without our being conscious of the slightest effort. But there comes a time when this effort becomes irksome without our knowing exactly what the matter is, only we know that instead of holding anything up close to see it, we must push it away, and if we attempt to read with the paper where it was formerly held, the eyes get tired. Our theory of vision makes the explanation of this self-evident. The emmetropic eye still sees distant objects the same as ever, but refuses to accommodate itself to near objects as formerly. The lens, becoming stiff with age, refuses longer to obey the ciliary muscle as once it did and no longer becomes so converse that its focus for near objects is on the retina.

The trouble need only be stated to suggest the remedy. If the lens will not thicken up at the centre and become more convex when we want to look at any thing close at hand, just put a piece of glass that is thicker at the centre than at the edges, in front of it, and thus reinforced, it performs its lessened work with ease and comfort as before. Now, this condition of the eye, when, through age, accommodation fails, is called the old eye or presbyopia. Theoretically, the relief of presbyopia by glasses is very simple, and in practice we find that such is the power of the eye to adapt itself to circumstances that very many presbyopic people use glasses which are not adapted to their eyes without much complaint. But very many people are complaining of weak eyes when the fault is not with the eyes but with badly adjusted spectacles. When, therefore, we attempt to adjust glasses to the presbyopic age, we find that there are several points to be considered. In the first place we find that the eye does not do its work with ease, unless it uses a certain part ot its accommodative power, e. g., suppose we want to give a man, fifty years of age, glasses with which to read at twelve inches distance from his eyes. A glass of twelve inches focus would throw the picture of this object upon the retina without any effort of the accommodation, but in practice the eye will not tolerate so strong a glass and wants a weaker one which will make the eye use some accommodation for perfect vision at this instance. Again, there is a curious relation between the amount of convergence of the two eyes, and the amount of accommodative effort which they can make. We may say that the accommodating power varies directly with the convergence, so that the nearer an object is held to the eyes, the greater is the convergence of the two eyes and the greater the power of accommodation. This fact must be borne in mind further on when we speak of glasses both for old people and for near-sighted people. There is one mere fact which experience has taught us, which we must consider in choosing the proper lens for any eye and that is that the eye cannot long use the whole of its accommodation without fatigue. That is to say, e. g., a man of forty-five can still accommodate for an object nine inches distant from his eye, but no closer; now he cannot read a print so fine that it has to be held at nine inches from his eye without great fatigue. While the youth, who can accommodate for a point five inches away, can read at nine inches with perfect ease. The principle is easy to see. It is difficult to work continuously up to one's limit of one's powers; intense fatigue soon results. So eyes which are just able to accommodate for nine inches by exerting the full force of the ciliary muscle, cannot continue to do so for any length of time without great fatigue. Having found for a patient lenses complying with the foregoing principles, the next question is, what kind of bows to use to keep them in front of his eyes. The first requisite of spectacle bows is that they shall hold the lenses steady and with their centres directly in front of the pupils. This is a very important point, and one to which too little attention is paid. The optician who sells spectacles ought to fix the frames to the shape of the features, as the tailor ought to fit us in coats or the shoemaker in shoes. But most of them pay no attention to this question; so the physician should investigate the fit of the spectacles whenever he sees his patients wearing them, even though they make no complaints.

A moment's study of the action of a lens upon the rays of light that pass through it, will show the reason for this. As the edge of a convex lens is thinner than the centre, the effect of looking through one side of the lens instead of through the centre, is the same as though we looked through a prism, and the object looked at seems moved out of place. You will remember that, in looking through a prism, the objects seem displaced in the direction of the thin edge of the prism; so in looking through a convex lens between the centre and the circumference, the object looked at will seem to be moved away from the centre towards the circumference of the lens. Now, if the spectacle bows are bent, as we frequently see them, so that one lens sets above the line of the pupils, an object as looked at through this lens looks to be lower than its true position; if the other lens is below the line of the pupils, so that the person is looking through its upper edge, the same object looks to be above its true position. Now, if one eye continues to see an object above its true position, and the other sees it below, we apparently see two objects instead of one, and this double vision the eye abhors so that the superior rectus muscle of one eye gives an extra pull to bring one object right and the inferior rectus of the other pulls the eye down to bring the other object right and the consequence of this discordant action is that the eyes soon become so wearied that work is impossible. The same thing holds true if the bows are so short that the centres of the lenses are nearer together than the pupils; or, if they are so long that the centres of the lenses are farther apart than the pupils. In the first place, when the lenses are too near together, each pupil looks through the outer edge of the lens, in consequence the object seems to be thrown to the right for the right eye, and to the left for the left eye, and the external recti have to pull the pupils apart or make the eyes diverge to prevent double images. Now you may ask what difference this makes, for the convergence of the eyes has to vary with the distance of the object anyway, so what matters a little more divergence than usual for a near object? You will remember that I spoke a few minutes ago of the relations between the convergence of the eyes and the accommodation, that the accommodation power was greater the greater the convergence. Now, if an object is thrown to the right by a glass before the right eye, which is so near the nose that the pupil, instead of being behind its centre, is behind its outside edge, and the same object is thrown to the left for the left eye in a similar manner, these eyes are made to diverge so much to bring these objects together and avoid double vision that their accommodative power is lessened, and this causes indistinct vision, weariness, etc., etc. So we see that the perfect spectacle frame will hold the lenses directly in front of the eyes, so that their centres will correspond with the centres of the pupils. This indication can hardly be met by any of the numerous patterns of eye-glasses now in the market, but they are so convenient to carry and to slip onto the nose when wanted for a short time, that their use is permissible under such circumstances, but for continuous use spectacles only should be used. For ordinary use the bows with a single side-piece, called single temples, are most convenient and, if rightly adjusted, hold the glasses with sufficient steadiness.

As for the lens itself, the choice lies between pebbles and good glass, with something to be said in favor of each. Glass is cheaper and some opticians say that being a little softer is more likely to be ground with perfect accuracy. But on account of their softness, glass lenses become scratched and rough sooner than pebbles. The advantages of pebbles are their superior hardness, which enables them to keep their polish longer, in careless hands. But spectacles are fragile, and usually are broken before they become scratched up, and the price of one pair of pebbles will buy several pairs of glass lenses. As for their effect upon the eyes there is absolutely no difference between them so far as I know.

So we see that our typical spectacles for age consist of well polished, accurately ground lenses, of such power that the person can see distinctly for a little while at a shorter distance than than that at which he wishes to hold his work; these lenses to be in a steady frame which holds the centres of the lenses in front of the pupils.

In myopia the conditions differ. Here, on account of the elongated eye-ball, the focus of the lens falls within the globe of the eye, and consequently but a very indistinct picture of distant objects falls upon the retina, while near objects can be seen with distinctness. Unfortunately, the eye does not possess any power the converse of accommodation, by which it can make the lens weaker and thus throw its image back upon the retina. So this has always to be supplied by external means. This, as we all know, consists of a concave lens, whose thick edges and thin centre have the effect of weakening the power of the crystalline lens and thus throwing its focus back upon the retina and making these a sharp image of the distant object. Then when the eye turns to a near object, it accommodates for it just as does the emmetropic eye. But here, too, we find that empirical facts come in to modify our theoretical conclusions. Myopia in childhood is a progressive disease and is likely to be followed by results very disastrous to vision, and this must be kept in mind in all dealings with near-sighted eyes. The tendency in myopia is toward a gradual distension of the tunics of the eye at its posterior pole, and this may cause such disorganization of retina and choroid as to impair the sensitiveness of the retina to visual impressions. This subject as a whole is far too wide a one to enter upon here. Our purpose is to consider only its practical aspect in relation to spectacles, and the first practical point which I would make is that in myopia properly fitted glasses exercise a decidedly salutary effect, in retarding or stopping the progress of this posterior bulging. The reason is this: myopic people are obliged to hold work and print so close to their eyes as to cause great convergence of the optical axes. Now this convergence is made by a strong contraction of the internal recti muscles, and the other ocular muscles take hold with a steady pull to keep the eyes from wavering about. The consequence is that great force is brought to bear upon the eye-balls, about the insertion of the recti muscles, and this acting upon the semi-fluid contents of the eye causes great pressure from within outwards at the posterior pole, which constantly tends to increase the existing distension. Suitable concave glasses oblige the wearer to remove the work farther from the eyes and thus relieve the strain from excessive convergence.

Another salutary effect of concave glasses in myopia is that they preserve the power of accommodation, which, without them is weakened or lost through disuse. The philosophy of this lies right on the surface. We will suppose a person is so short-sighted that objects twelve inches from his face are pictured upon the retina without any use of the ciliary muscle in accommodation, you will see at once that this ciliary muscle will very rarely be called into action, for we seldom need to bring anything nearer than twelve inches from the eye to see it, and for anything beyond this distance, of course, the ciliary muscle and accommodation would be entirely relaxed. The consequence is the same here as anywhere else where muscles are kept idle. This muscle loses tone and power, and after a time can be used at all only with difficulty and fatigue. Now, if in youth, before disuse has effected this ciliary muscle, we put a concave glass of twelve inches focus before there eyes, the picture of distant objects falls directly upon the retina; to see anything nearer accommodation comes into play, and so the ciliary muscle being constantly exercised, its power and tone are preserved in a normal condition. If you will pardon a bit of autobiography, I will illustrate this point by my own case. When I was a lad, twelve or fourteen years old, I found I could not see the blackboard in school so as to learn from it what others did, and I bought me a pair of glasses. Every one said I would ruin my eyes by wearing glasses, but I could see distinctly with them and could not see at a distance without them, and so I wore them and have worn them constantly from that time till now. My myopia has not increased any since I was seventeen years of age, and I have kept my accommodation in perfect working order.

Another imperative reason why the myopic person should put on spectacles is, that they may see what goes on around them as well as other people. It is a terrible weight in the fierce race of life to be unable to see with distinctness anything beyond an arms length from one's face. In order to judge of the myope's condition, just put on a pair of convex glasses, say from twelve to eighteen inches focal distance, then you will see things about as an average near-sighted person sees them. Faces will lose all expression; animals, birds, flowers, trees become simply a blotch of color. Nothing can be learned from observation, because nothing can be distinguished and the unfortunate is driven to books as a solace, whose comforting pages only aggravate the difficulty which leads one to seek their consolation.

As myopic people usually wear glasses all the time, I think that bows which hook behind the ears, are preferable, as they hold the lenses steadier than single temples. As concave lenses are thicker at the edges than in the centre, it is easy to groove the edge to let in the bows and this prevents them from slipping out. Light steel bows are ordinarily chosen in this latitude at the sea shore, however, and in warmer, damper climates, steel rusts out very soon and the bows break at the bridge; so some avocations expose a person to steam and gases, which rust steel. In these cases gold bows are more economical, although the first cost is greater. Personally I prefer a frame without a stop at the joint, as being more durable and less likely to be bent by any accidental blow. After twenty-five years experience and many experiments I have adopted the style of bows which I now wear as the typical bow for constant use. But these hooked bows are never right as they come from the shops. The hooks are bent in a circle of too great radius and the bend begins too near to the lens. The side-piece should be straight until it reaches the top of the ear and then bend in a small circle to fit close behind it. I would never advise pebbles for concave glasses. The thick edges keep the surface of the glass from touching, when laid down or put into a case so that it is not so liable to be scratched as a convex glass, and opticians are apt to chip and deface the hard pebbles when grooving them to fit a neat frame. Extra care should be taken in glasses for continuous use that the centres of the lenses coincide with spectacles the position of the pupils, for reasons which I have before mentioned. For high degrees of myopia it is advisable to have two pairs of glasses, one for distance which perfectly neutralizes the myopia, and for close work a little weaker. But such cases come legitimately under the care of a specialist and need not be considered here.

I have already trespassed long on your patience and I have not reached the other errors of refraction which demand spectacles, hypermetropia and astigmatism. Suffice it to say that there are multitudes of cases of weak, irritable, inflamed eyes, which are caused by the strain which attends upon any use of eyes affected by these refractive errors whose existence is not suspected, and very many of these cases could be restored to perfect functions by the use of properly adjusted convex or cylindrical lenses.

